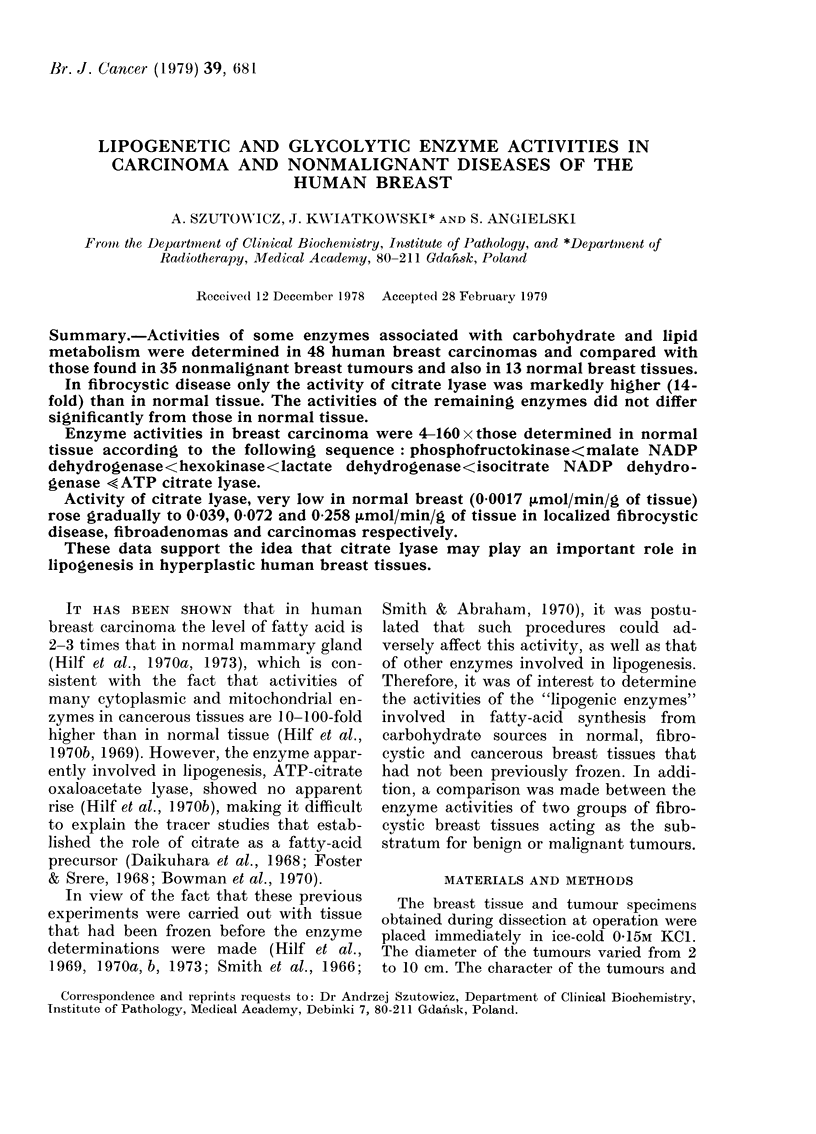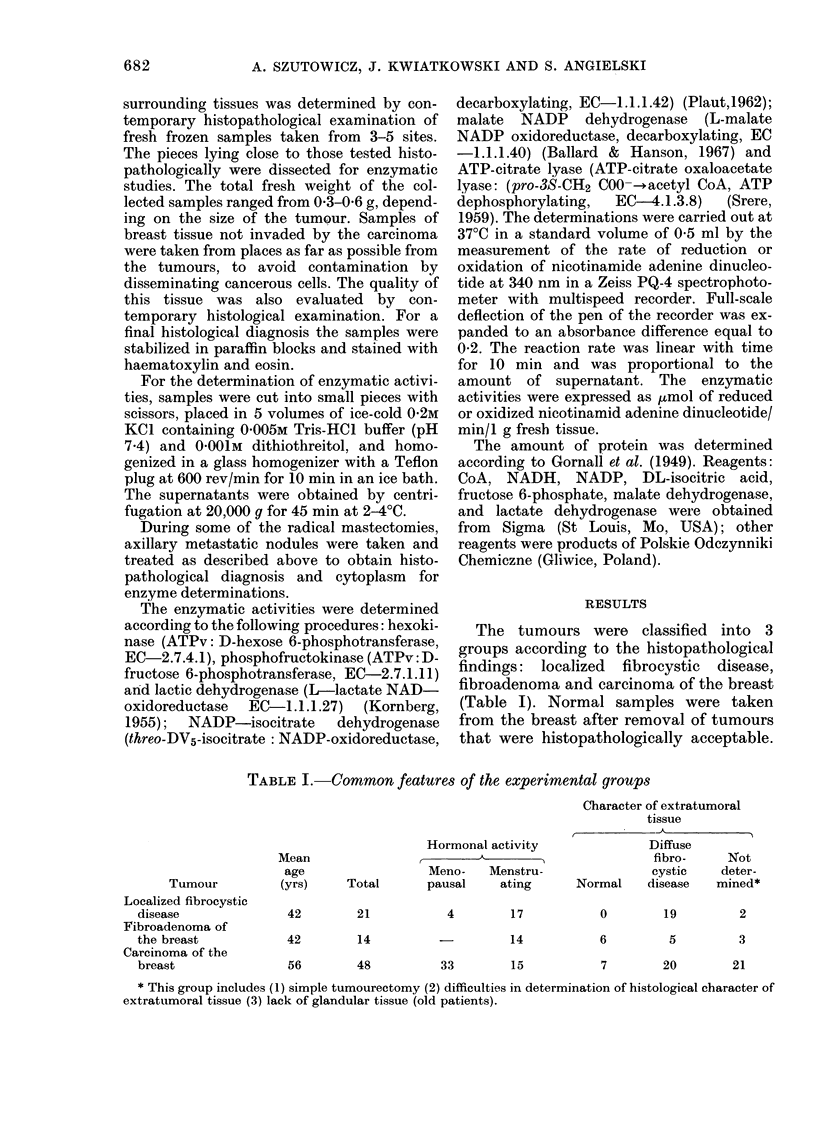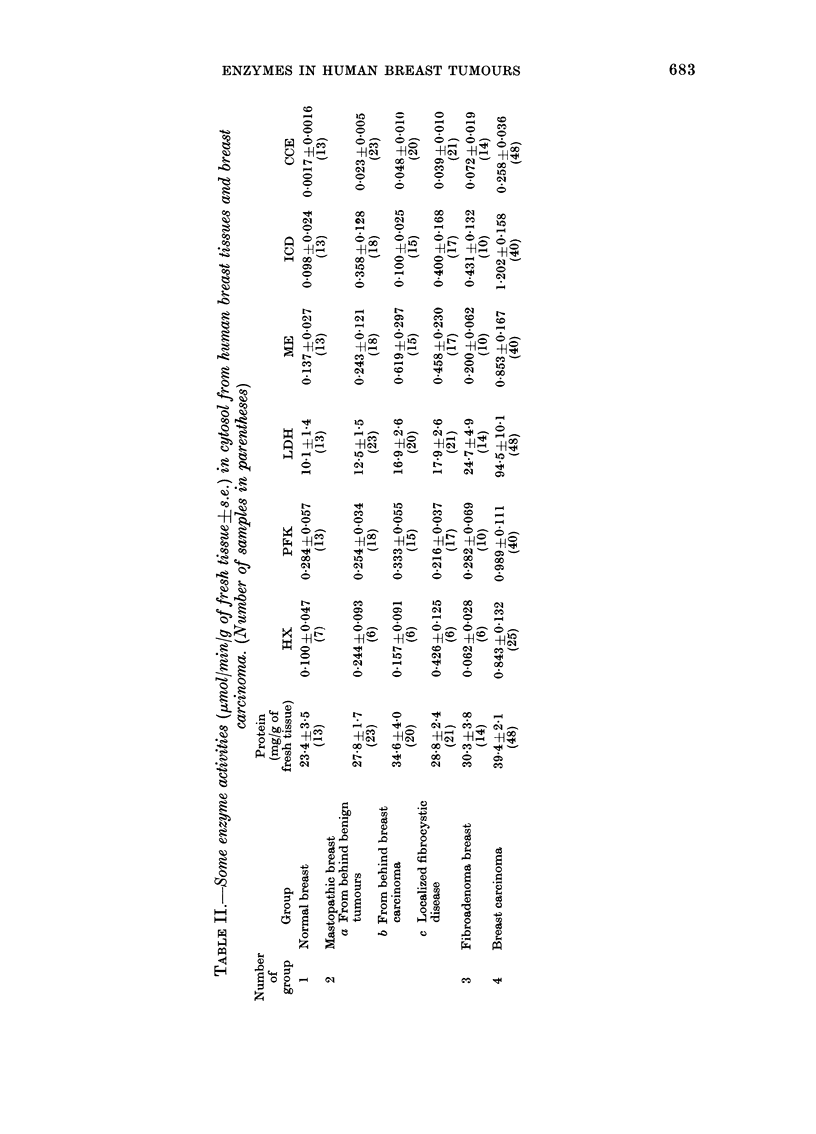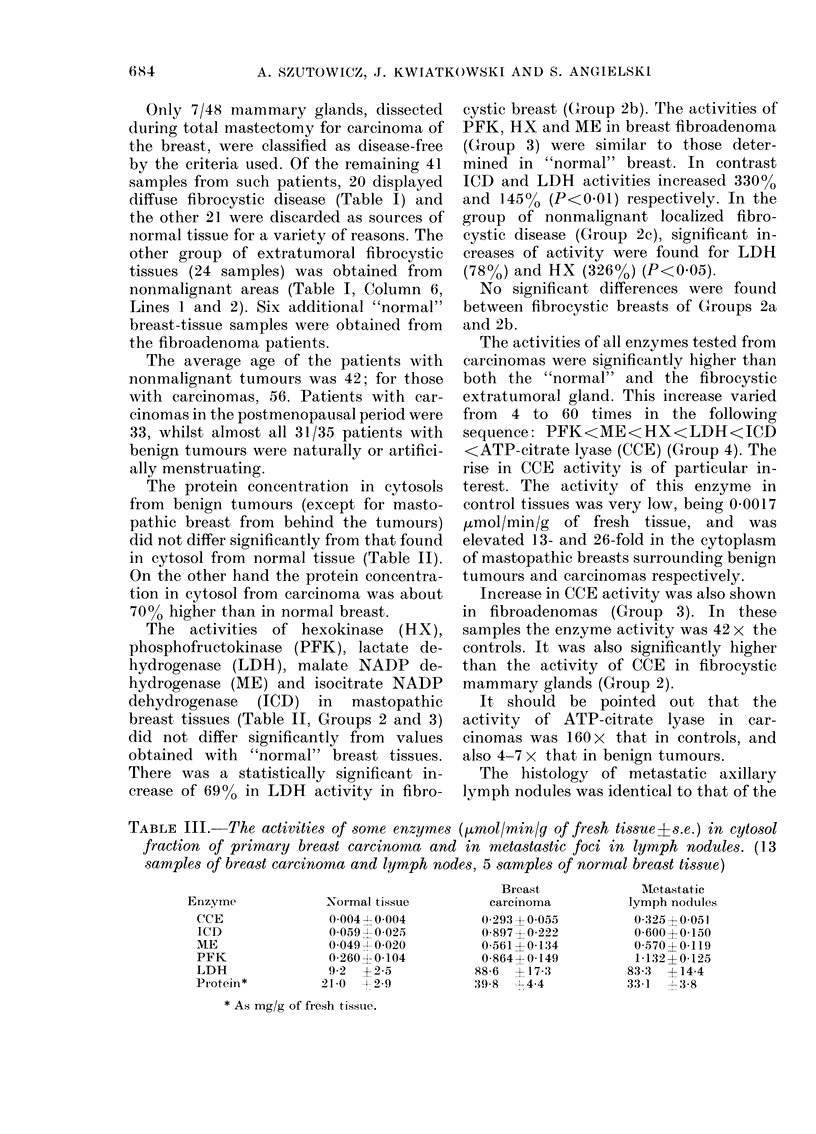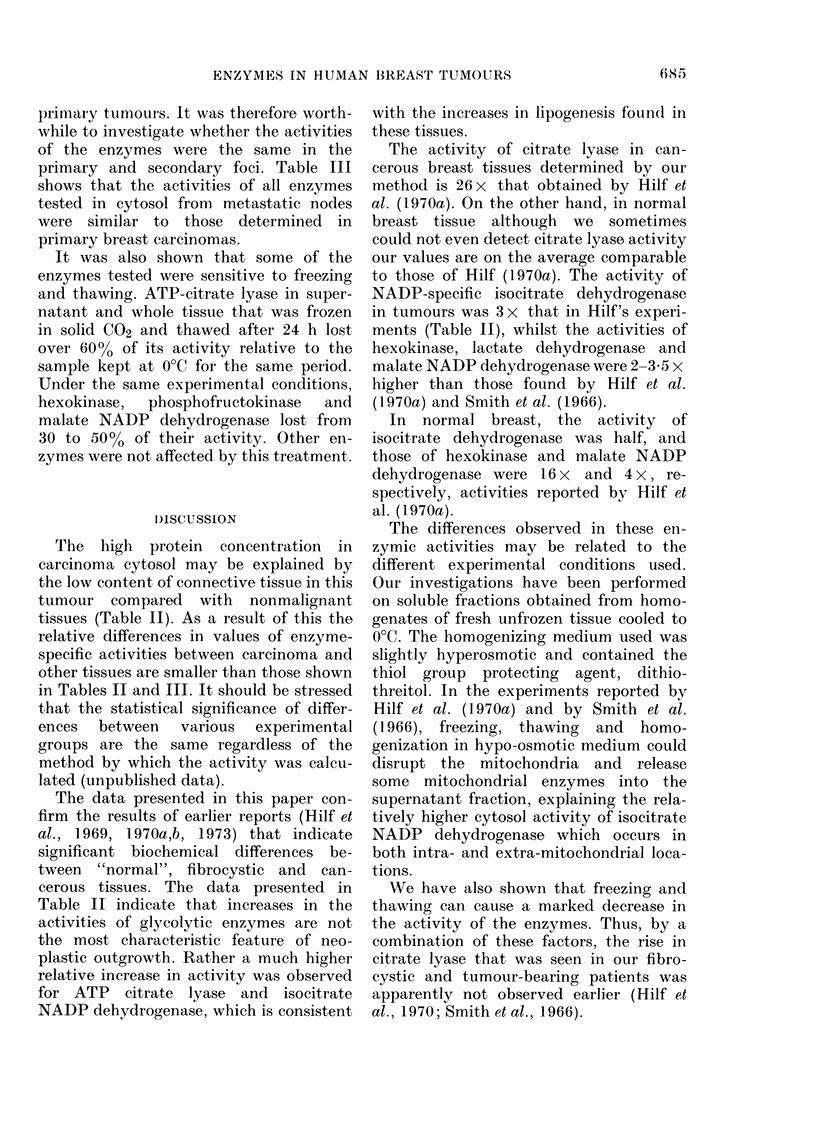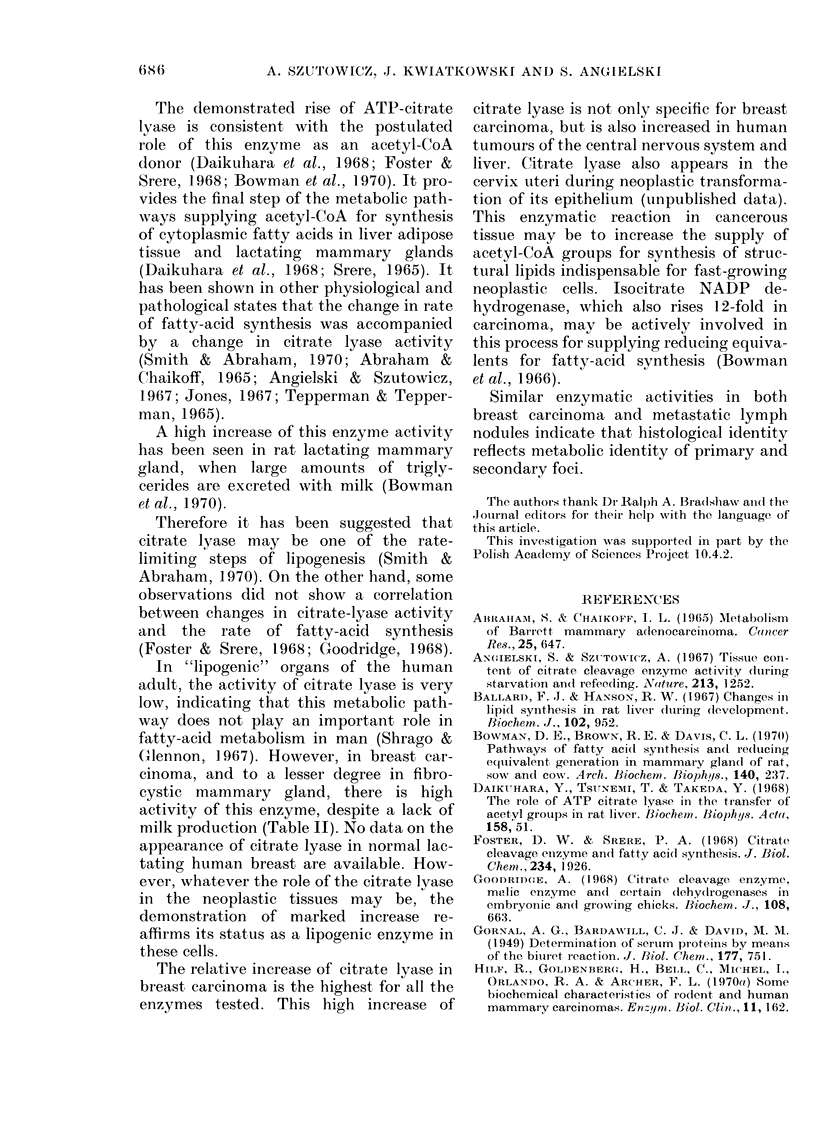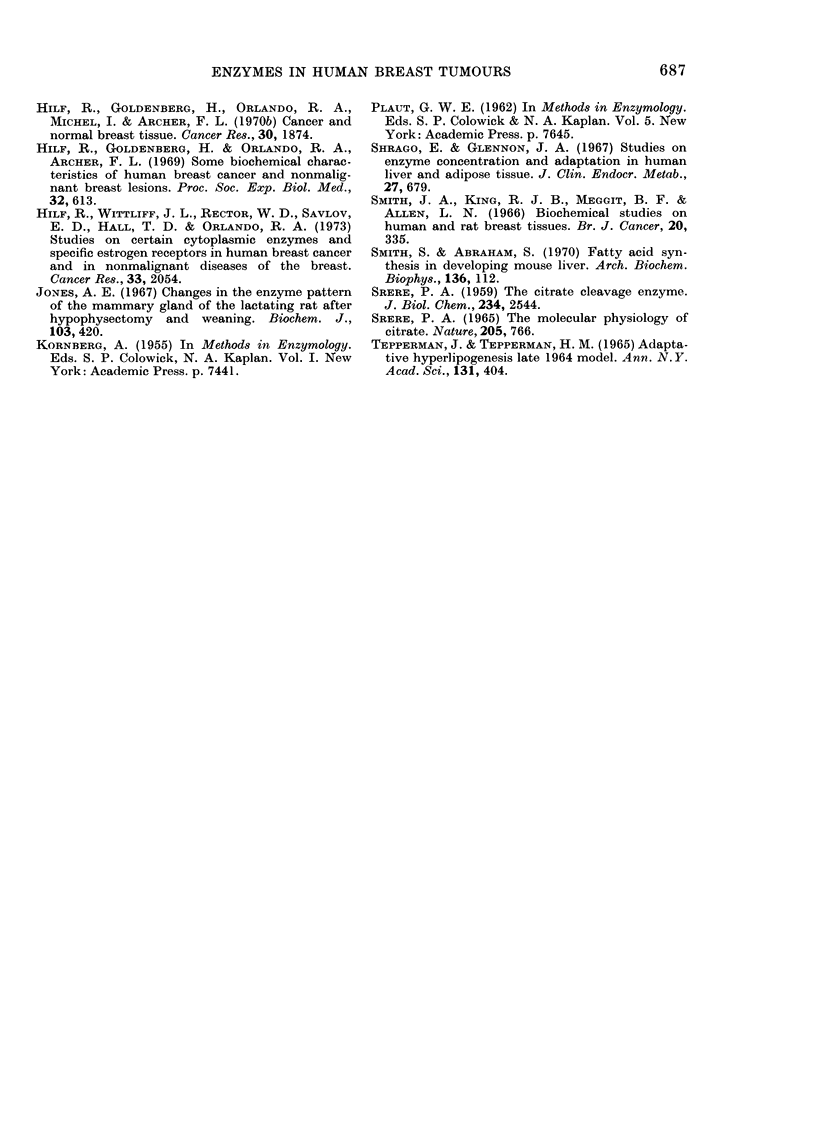# Lipogenetic and glycolytic enzyme activities in carcinoma and nonmalignant diseases of the human breast.

**DOI:** 10.1038/bjc.1979.120

**Published:** 1979-06

**Authors:** A. Szutowicz, J. Kwiatkowski, S. Angielski

## Abstract

Activities of some enzymes associated with carbohydrate and lipid metabolism were determined in 48 human breast carcinomas and compared with those found in 35 nonmalignant breast tumours and also in 13 normal breast tissues. In fibrocystic disease only the activity of citrate lyase was markedly higher (14-fold) than in normal tissue. The activities of the remaining enzymes did not differ significantly from those in normal tissue. Enzyme activities in breast carcinoma were 4--160 x those determined in normal tissue according to the following sequence : phosphofructokinase less than malate NADP dehydrogenase less than hexokinase less than lactate dehydrogenase less than isocitrate NADP dehydrogenase less than ATP citrate lyase. Activity of citrate lyase, very low in normal breast (0.0017 mumol/min/g of tissue) rose gradually to 0.039, 0.072 and 0.258 mumol/min/g of tissue in localized fibrocystic disease, fibroadenomas and carcinomas respectively. These data support the idea that citrate lyase may play an important role in lipogenesis in hyperplastic human breast tissues.


*Br. J. Cancer* (1979) **39**, 681

### LIPOGENETIC AND GLYCOLYTIC ENZYME ACTIVITIES IN CARCINOMA AND NONMALIGNANT DISEASES OF THE HUMAN BREAST

A. SZUTOWICZ, J. KWIATKOWSKI* AND S. ANGIELSKI

*From the Department of Clinical Biochemistry, Institute of Pathology, and *Department of Radiotherapy, Medical Academy, 80–211 Gdańsk, Poland*

Received 12 December 1978 Accepted 28 February 1979

**Summary.—Activities of some enzymes associated with carbohydrate and lipid metabolism were determined in 48 human breast carcinomas and compared with those found in 35 nonmalignant breast tumours and also in 13 normal breast tissues.**

**In fibrocystic disease only the activity of citrate lyase was markedly higher (14-fold) than in normal tissue. The activities of the remaining enzymes did not differ significantly from those in normal tissue.**

**Enzyme activities in breast carcinoma were 4–160 × those determined in normal tissue according to the following sequence : phosphofructokinase < malate NADP dehydrogenase < hexokinase < lactate dehydrogenase < isocitrate NADP dehydrogenase ≪ ATP citrate lyase.**

**Activity of citrate lyase, very low in normal breast (0·0017 μmol/min/g of tissue) rose gradually to 0·039, 0·072 and 0·258 μmol/min/g of tissue in localized fibrocystic disease, fibroadenomas and carcinomas respectively.**

**These data support the idea that citrate lyase may play an important role in lipogenesis in hyperplastic human breast tissues.**

IT HAS BEEN SHOWN that in human breast carcinoma the level of fatty acid is 2–3 times that in normal mammary gland (Hilf *et al.*, 1970*a*, 1973), which is consistent with the fact that activities of many cytoplasmic and mitochondrial enzymes in cancerous tissues are 10–100-fold higher than in normal tissue (Hilf *et al.*, 1970*b*, 1969). However, the enzyme apparently involved in lipogenesis, ATP-citrate oxaloacetate lyase, showed no apparent rise (Hilf *et al.*, 1970*b*), making it difficult to explain the tracer studies that established the role of citrate as a fatty-acid precursor (Daikuhara *et al.*, 1968; Foster & Srere, 1968; Bowman *et al.*, 1970).

In view of the fact that these previous experiments were carried out with tissue that had been frozen before the enzyme determinations were made (Hilf *et al.*, 1969, 1970*a*, *b*, 1973; Smith *et al.*, 1966; Smith & Abraham, 1970), it was postulated that such procedures could adversely affect this activity, as well as that of other enzymes involved in lipogenesis. Therefore, it was of interest to determine the activities of the "lipogenic enzymes" involved in fatty-acid synthesis from carbohydrate sources in normal, fibrocystic and cancerous breast tissues that had not been previously frozen. In addition, a comparison was made between the enzyme activities of two groups of fibrocystic breast tissues acting as the substratum for benign or malignant tumours.

### MATERIALS AND METHODS

The breast tissue and tumour specimens obtained during dissection at operation were placed immediately in ice-cold 0·15M KCl. The diameter of the tumours varied from 2 to 10 cm. The character of the tumours and

Correspondence and reprints requests to: Dr Andrzej Szutowicz, Department of Clinical Biochemistry, Institute of Pathology, Medical Academy, Debinki 7, 80-211 Gdańsk, Poland.

surrounding tissues was determined by contemporary histopathological examination of fresh frozen samples taken from 3–5 sites. The pieces lying close to those tested histopathologically were dissected for enzymatic studies. The total fresh weight of the collected samples ranged from 0·3–0·6 g, depending on the size of the tumour. Samples of breast tissue not invaded by the carcinoma were taken from places as far as possible from the tumours, to avoid contamination by disseminating cancerous cells. The quality of this tissue was also evaluated by contemporary histological examination. For a final histological diagnosis the samples were stabilized in paraffin blocks and stained with haematoxylin and eosin.

For the determination of enzymatic activities, samples were cut into small pieces with scissors, placed in 5 volumes of ice-cold 0·2M KCl containing 0·005M Tris-HCl buffer (pH 7·4) and 0·001M dithiothreitol, and homogenized in a glass homogenizer with a Teflon plug at 600 rev/min for 10 min in an ice bath. The supernatants were obtained by centrifugation at 20,000 *g* for 45 min at 2–4°C.

During some of the radical mastectomies, axillary metastatic nodules were taken and treated as described above to obtain histopathological diagnosis and cytoplasm for enzyme determinations.

The enzymatic activities were determined according to the following procedures: hexokinase (ATPv: D-hexose 6-phosphotransferase, EC—2.7.4.1), phosphofructokinase (ATPv: D-fructose 6-phosphotransferase, EC—2.7.1.11) and lactic dehydrogenase (L—lactate NAD—oxidoreductase EC—1.1.1.27) (Kornberg, 1955); NADP—isocitrate dehydrogenase (*threo*-$DV_5$-isocitrate : NADP-oxidoreductase, decarboxylating, EC—1.1.1.42) (Plaut,1962); malate NADP dehydrogenase (L-malate NADP oxidoreductase, decarboxylating, EC —1.1.1.40) (Ballard & Hanson, 1967) and ATP-citrate lyase (ATP-citrate oxaloacetate lyase: (*pro-3S*-$CH_2$ $COO^-$→acetyl CoA, ATP dephosphorylating, EC—4.1.3.8) (Srere, 1959). The determinations were carried out at 37°C in a standard volume of 0·5 ml by the measurement of the rate of reduction or oxidation of nicotinamide adenine dinucleotide at 340 nm in a Zeiss PQ-4 spectrophotometer with multispeed recorder. Full-scale deflection of the pen of the recorder was expanded to an absorbance difference equal to 0·2. The reaction rate was linear with time for 10 min and was proportional to the amount of supernatant. The enzymatic activities were expressed as μmol of reduced or oxidized nicotinamid adenine dinucleotide/min/1 g fresh tissue.

The amount of protein was determined according to Gornall *et al.* (1949). Reagents: CoA, NADH, NADP, DL-isocitric acid, fructose 6-phosphate, malate dehydrogenase, and lactate dehydrogenase were obtained from Sigma (St Louis, Mo, USA); other reagents were products of Polskie Odczynniki Chemiczne (Gliwice, Poland).

### RESULTS

The tumours were classified into 3 groups according to the histopathological findings: localized fibrocystic disease, fibroadenoma and carcinoma of the breast (Table I). Normal samples were taken from the breast after removal of tumours that were histopathologically acceptable.

TABLE I.—*Common features of the experimental groups*

| Tumour | Mean age (yrs) | Total | Hormonal activity: Menopausal | Hormonal activity: Menstruating | Character of extratumoral tissue: Normal | Character of extratumoral tissue: Diffuse fibrocystic disease | Character of extratumoral tissue: Not determined* |
|---|---|---|---|---|---|---|---|
| Localized fibrocystic disease | 42 | 21 | 4 | 17 | 0 | 19 | 2 |
| Fibroadenoma of the breast | 42 | 14 | — | 14 | 6 | 5 | 3 |
| Carcinoma of the breast | 56 | 48 | 33 | 15 | 7 | 20 | 21 |

* This group includes (1) simple tumourectomy (2) difficulties in determination of histological character of extratumoral tissue (3) lack of glandular tissue (old patients).

TABLE II.—*Some enzyme activities (μmol/min/g of fresh tissue ± s.e.) in cytosol from human breast tissues and breast carcinoma. (Number of samples in parentheses)*

| Number of group | Group | Protein (mg/g of fresh tissue) | HX | PFK | LDH | ME | ICD | CCE |
|---|---|---|---|---|---|---|---|---|
| 1 | Normal breast | 23·4 ± 3·5 (13) | 0·100 ± 0·047 (7) | 0·284 ± 0·057 (13) | 10·1 ± 1·4 (13) | 0·137 ± 0·027 (13) | 0·098 ± 0·024 (13) | 0·0017 ± 0·0016 (13) |
| 2 | Mastopathic breast | | | | | | | |
| | *a* From behind benign tumours | 27·8 ± 1·7 (23) | 0·244 ± 0·093 (6) | 0·254 ± 0·034 (18) | 12·5 ± 1·5 (23) | 0·243 ± 0·121 (18) | 0·358 ± 0·128 (18) | 0·023 ± 0·005 (23) |
| | *b* From behind breast carcinoma | 34·6 ± 4·0 (20) | 0·157 ± 0·091 (6) | 0·333 ± 0·055 (15) | 16·9 ± 2·6 (20) | 0·619 ± 0·297 (15) | 0·100 ± 0·025 (15) | 0·048 ± 0·010 (20) |
| | *c* Localized fibrocystic disease | 28·8 ± 2·4 (21) | 0·426 ± 0·125 (6) | 0·216 ± 0·037 (17) | 17·9 ± 2·6 (21) | 0·458 ± 0·230 (17) | 0·400 ± 0·168 (17) | 0·039 ± 0·010 (21) |
| 3 | Fibroadenoma breast | 30·3 ± 3·8 (14) | 0·062 ± 0·028 (6) | 0·282 ± 0·069 (10) | 24·7 ± 4·9 (14) | 0·200 ± 0·062 (10) | 0·431 ± 0·132 (10) | 0·072 ± 0·019 (14) |
| 4 | Breast carcinoma | 39·4 ± 2·1 (48) | 0·843 ± 0·132 (25) | 0·989 ± 0·111 (40) | 94·5 ± 10·1 (48) | 0·853 ± 0·167 (40) | 1·202 ± 0·158 (40) | 0·258 ± 0·036 (48) |

Only 7/48 mammary glands, dissected during total mastectomy for carcinoma of the breast, were classified as disease-free by the criteria used. Of the remaining 41 samples from such patients, 20 displayed diffuse fibrocystic disease (Table I) and the other 21 were discarded as sources of normal tissue for a variety of reasons. The other group of extratumoral fibrocystic tissues (24 samples) was obtained from nonmalignant areas (Table I, Column 6, Lines 1 and 2). Six additional "normal" breast-tissue samples were obtained from the fibroadenoma patients.

The average age of the patients with nonmalignant tumours was 42; for those with carcinomas, 56. Patients with carcinomas in the postmenopausal period were 33, whilst almost all 31/35 patients with benign tumours were naturally or artificially menstruating.

The protein concentration in cytosols from benign tumours (except for mastopathic breast from behind the tumours) did not differ significantly from that found in cytosol from normal tissue (Table II). On the other hand the protein concentration in cytosol from carcinoma was about 70% higher than in normal breast.

The activities of hexokinase (HX), phosphofructokinase (PFK), lactate dehydrogenase (LDH), malate NADP dehydrogenase (ME) and isocitrate NADP dehydrogenase (ICD) in mastopathic breast tissues (Table II, Groups 2 and 3) did not differ significantly from values obtained with "normal" breast tissues. There was a statistically significant increase of 69% in LDH activity in fibrocystic breast (Group 2b). The activities of PFK, HX and ME in breast fibroadenoma (Group 3) were similar to those determined in "normal" breast. In contrast ICD and LDH activities increased 330% and 145% ($P<0{\cdot}01$) respectively. In the group of nonmalignant localized fibrocystic disease (Group 2c), significant increases of activity were found for LDH (78%) and HX (326%) ($P<0{\cdot}05$).

No significant differences were found between fibrocystic breasts of Groups 2a and 2b.

The activities of all enzymes tested from carcinomas were significantly higher than both the "normal" and the fibrocystic extratumoral gland. This increase varied from 4 to 60 times in the following sequence: PFK<ME<HX<LDH<ICD <ATP-citrate lyase (CCE) (Group 4). The rise in CCE activity is of particular interest. The activity of this enzyme in control tissues was very low, being 0·0017 μmol/min/g of fresh tissue, and was elevated 13- and 26-fold in the cytoplasm of mastopathic breasts surrounding benign tumours and carcinomas respectively.

Increase in CCE activity was also shown in fibroadenomas (Group 3). In these samples the enzyme activity was 42× the controls. It was also significantly higher than the activity of CCE in fibrocystic mammary glands (Group 2).

It should be pointed out that the activity of ATP-citrate lyase in carcinomas was 160× that in controls, and also 4–7× that in benign tumours.

The histology of metastatic axillary lymph nodules was identical to that of the

TABLE III.—*The activities of some enzymes (μmol/min/g of fresh tissue±s.e.) in cytosol fraction of primary breast carcinoma and in metastastic foci in lymph nodules.* (13 *samples of breast carcinoma and lymph nodes,* 5 *samples of normal breast tissue*)

| Enzyme | Normal tissue | Breast carcinoma | Metastatic lymph nodules |
|---|---|---|---|
| CCE | 0·004±0·004 | 0·293±0·055 | 0·325±0·051 |
| ICD | 0·059±0·025 | 0·897±0·222 | 0·600±0·150 |
| ME | 0·049±0·020 | 0·561±0·134 | 0·570±0·119 |
| PFK | 0·260±0·104 | 0·864±0·149 | 1·132±0·125 |
| LDH | 9·2 ±2·5 | 88·6 ±17·3 | 83·3 ±14·4 |
| Protein* | 21·0 ±2·9 | 39·8 ±4·4 | 33·1 ±3·8 |

* As mg/g of fresh tissue.

primary tumours. It was therefore worthwhile to investigate whether the activities of the enzymes were the same in the primary and secondary foci. Table III shows that the activities of all enzymes tested in cytosol from metastatic nodes were similar to those determined in primary breast carcinomas.

It was also shown that some of the enzymes tested were sensitive to freezing and thawing. ATP-citrate lyase in supernatant and whole tissue that was frozen in solid $CO_2$ and thawed after 24 h lost over 60% of its activity relative to the sample kept at 0°C for the same period. Under the same experimental conditions, hexokinase, phosphofructokinase and malate NADP dehydrogenase lost from 30 to 50% of their activity. Other enzymes were not affected by this treatment.

### DISCUSSION

The high protein concentration in carcinoma cytosol may be explained by the low content of connective tissue in this tumour compared with nonmalignant tissues (Table II). As a result of this the relative differences in values of enzyme-specific activities between carcinoma and other tissues are smaller than those shown in Tables II and III. It should be stressed that the statistical significance of differences between various experimental groups are the same regardless of the method by which the activity was calculated (unpublished data).

The data presented in this paper confirm the results of earlier reports (Hilf *et al.*, 1969, 1970*a*,*b*, 1973) that indicate significant biochemical differences between "normal", fibrocystic and cancerous tissues. The data presented in Table II indicate that increases in the activities of glycolytic enzymes are not the most characteristic feature of neoplastic outgrowth. Rather a much higher relative increase in activity was observed for ATP citrate lyase and isocitrate NADP dehydrogenase, which is consistent with the increases in lipogenesis found in these tissues.

The activity of citrate lyase in cancerous breast tissues determined by our method is 26× that obtained by Hilf *et al.* (1970*a*). On the other hand, in normal breast tissue although we sometimes could not even detect citrate lyase activity our values are on the average comparable to those of Hilf (1970*a*). The activity of NADP-specific isocitrate dehydrogenase in tumours was 3× that in Hilf's experiments (Table II), whilst the activities of hexokinase, lactate dehydrogenase and malate NADP dehydrogenase were 2–3·5× higher than those found by Hilf *et al.* (1970*a*) and Smith *et al.* (1966).

In normal breast, the activity of isocitrate dehydrogenase was half, and those of hexokinase and malate NADP dehydrogenase were 16× and 4×, respectively, activities reported by Hilf *et* al. (1970*a*).

The differences observed in these enzymic activities may be related to the different experimental conditions used. Our investigations have been performed on soluble fractions obtained from homogenates of fresh unfrozen tissue cooled to 0°C. The homogenizing medium used was slightly hyperosmotic and contained the thiol group protecting agent, dithiothreitol. In the experiments reported by Hilf *et al.* (1970*a*) and by Smith *et al.* (1966), freezing, thawing and homogenization in hypo-osmotic medium could disrupt the mitochondria and release some mitochondrial enzymes into the supernatant fraction, explaining the relatively higher cytosol activity of isocitrate NADP dehydrogenase which occurs in both intra- and extra-mitochondrial locations.

We have also shown that freezing and thawing can cause a marked decrease in the activity of the enzymes. Thus, by a combination of these factors, the rise in citrate lyase that was seen in our fibrocystic and tumour-bearing patients was apparently not observed earlier (Hilf *et al.*, 1970; Smith *et al.*, 1966).

The demonstrated rise of ATP-citrate lyase is consistent with the postulated role of this enzyme as an acetyl-CoA donor (Daikuhara *et al.*, 1968; Foster & Srere, 1968; Bowman *et al.*, 1970). It provides the final step of the metabolic pathways supplying acetyl-CoA for synthesis of cytoplasmic fatty acids in liver adipose tissue and lactating mammary glands (Daikuhara *et al.*, 1968; Srere, 1965). It has been shown in other physiological and pathological states that the change in rate of fatty-acid synthesis was accompanied by a change in citrate lyase activity (Smith & Abraham, 1970; Abraham & Chaikoff, 1965; Angielski & Szutowicz, 1967; Jones, 1967; Tepperman & Tepperman, 1965).

A high increase of this enzyme activity has been seen in rat lactating mammary gland, when large amounts of triglycerides are excreted with milk (Bowman *et al.*, 1970).

Therefore it has been suggested that citrate lyase may be one of the rate-limiting steps of lipogenesis (Smith & Abraham, 1970). On the other hand, some observations did not show a correlation between changes in citrate-lyase activity and the rate of fatty-acid synthesis (Foster & Srere, 1968; Goodridge, 1968).

In "lipogenic" organs of the human adult, the activity of citrate lyase is very low, indicating that this metabolic pathway does not play an important role in fatty-acid metabolism in man (Shrago & Glennon, 1967). However, in breast carcinoma, and to a lesser degree in fibrocystic mammary gland, there is high activity of this enzyme, despite a lack of milk production (Table II). No data on the appearance of citrate lyase in normal lactating human breast are available. However, whatever the role of the citrate lyase in the neoplastic tissues may be, the demonstration of marked increase reaffirms its status as a lipogenic enzyme in these cells.

The relative increase of citrate lyase in breast carcinoma is the highest for all the enzymes tested. This high increase of citrate lyase is not only specific for breast carcinoma, but is also increased in human tumours of the central nervous system and liver. Citrate lyase also appears in the cervix uteri during neoplastic transformation of its epithelium (unpublished data). This enzymatic reaction in cancerous tissue may be to increase the supply of acetyl-CoA groups for synthesis of structural lipids indispensable for fast-growing neoplastic cells. Isocitrate NADP dehydrogenase, which also rises 12-fold in carcinoma, may be actively involved in this process for supplying reducing equivalents for fatty-acid synthesis (Bowman *et al.*, 1966).

Similar enzymatic activities in both breast carcinoma and metastatic lymph nodules indicate that histological identity reflects metabolic identity of primary and secondary foci.

The authors thank Dr Ralph A. Bradshaw and the Journal editors for their help with the language of this article.

This investigation was supported in part by the Polish Academy of Sciences Project 10.4.2.

### REFERENCES

Abraham, S. & Chaikoff, I. L. (1965) Metabolism of Barrett mammary adenocarcinoma. *Cancer Res.*, **25**, 647.

Angielski, S. & Szutowicz, A. (1967) Tissue content of citrate cleavage enzyme activity during starvation and refeeding. *Nature*, **213**, 1252.

Ballard, F. J. & Hanson, R. W. (1967) Changes in lipid synthesis in rat liver during development. *Biochem. J.*, **102**, 952.

Bowman, D. E., Brown, R. E. & Davis, C. L. (1970) Pathways of fatty acid synthesis and reducing equivalent generation in mammary gland of rat, sow and cow. *Arch. Biochem. Biophys.*, **140**, 237.

Daikuhara, Y., Tsunemi, T. & Takeda, Y. (1968) The role of ATP citrate lyase in the transfer of acetyl groups in rat liver. *Biochem. Biophys. Acta*, **158**, 51.

Foster, D. W. & Srere, P. A. (1968) Citrate cleavage enzyme and fatty acid synthesis. *J. Biol. Chem.*, **234**, 1926.

Goodridge, A. (1968) Citrate cleavage enzyme, malic enzyme and certain dehydrogenases in embryonic and growing chicks. *Biochem. J.*, **108**, 663.

Gornal, A. G., Bardawill, C. J. & David, M. M. (1949) Determination of serum proteins by means of the biuret reaction. *J. Biol. Chem.*, **177**, 751.

Hilf, R., Goldenberg, H., Bell, C., Michel, I., Orlando, R. A. & Archer, F. L. (1970*a*) Some biochemical characteristics of rodent and human mammary carcinomas. *Enzym. Biol. Clin.*, **11**, 162.

HILF, R., GOLDENBERG, H., ORLANDO, R. A., MICHEL, I. & ARCHER, F. L. (1970*b*) Cancer and normal breast tissue. *Cancer Res.*, **30**, 1874.

HILF, R., GOLDENBERG, H. & ORLANDO, R. A., ARCHER, F. L. (1969) Some biochemical characteristics of human breast cancer and nonmalignant breast lesions. *Proc. Soc. Exp. Biol. Med.*, **32**, 613.

HILF, R., WITTLIFF, J. L., RECTOR, W. D., SAVLOV, E. D., HALL, T. D. & ORLANDO, R. A. (1973) Studies on certain cytoplasmic enzymes and specific estrogen receptors in human breast cancer and in nonmalignant diseases of the breast. *Cancer Res.*, **33**, 2054.

JONES, A. E. (1967) Changes in the enzyme pattern of the mammary gland of the lactating rat after hypophysectomy and weaning. *Biochem. J.*, **103**, 420.

KORNBERG, A. (1955) In *Methods in Enzymology*. Eds. S. P. Colowick, N. A. Kaplan. Vol. I. New York: Academic Press. p. 7441.

PLAUT, G. W. E. (1962) In *Methods in Enzymology*. Eds. S. P. Colowick & N. A. Kaplan. Vol. 5. New York: Academic Press. p. 7645.

SHRAGO, E. & GLENNON, J. A. (1967) Studies on enzyme concentration and adaptation in human liver and adipose tissue. *J. Clin. Endocr. Metab.*, **27**, 679.

SMITH, J. A., KING, R. J. B., MEGGIT, B. F. & ALLEN, L. N. (1966) Biochemical studies on human and rat breast tissues. *Br. J. Cancer*, **20**, 335.

SMITH, S. & ABRAHAM, S. (1970) Fatty acid synthesis in developing mouse liver. *Arch. Biochem. Biophys.*, **136**, 112.

SRERE, P. A. (1959) The citrate cleavage enzyme. *J. Biol. Chem.*, **234**, 2544.

SRERE, P. A. (1965) The molecular physiology of citrate. *Nature*, **205**, 766.

TEPPERMAN, J. & TEPPERMAN, H. M. (1965) Adaptative hyperlipogenesis late 1964 model. *Ann. N.Y. Acad. Sci.*, **131**, 404.